# Formal comment on “Systematic review of the predictors of statin adherence for the primary prevention of cardiovascular disease”

**DOI:** 10.1371/journal.pone.0205138

**Published:** 2019-01-17

**Authors:** David M. Diamond, Michel de Lorgeril, Malcolm Kendrick, Uffe Ravnskov, Paul J. Rosch

**Affiliations:** 1 Departments of Psychology, University of South Florida, Tampa, FL, United States of America; 2 Molecular Pharmacology & Physiology, University of South Florida, Tampa, FL, United States of America; 3 Laboratoire Coeur et Nutrition, TIMC-IMAG, School of Medicine, University of Grenoble-Alpes, Grenoble, France; 4 East Cheshire Trust, Macclesfield District General Hospital, Macclesfield, Cheshire, United Kingdom; 5 Magle Stora Kyrkogata, Lund, Sweden; 6 New York Medical College; Valhalla, New York; 7 The American Institute of Stress, Fort Worth, TX; Leibniz Institute for Prevention Research and Epidemiology BIPS, GERMANY

## Abstract

Statins have been prescribed for primary prevention of cardiovascular disease (CVD) for nearly 3 decades. Throughout this period key opinion leaders in the field have been dismayed by the high rate of non-adherence of patients to follow their statin regimen. Hope et al., [1] have addressed this issue by providing a systematic review of research on predictors of statin adherence for primary prevention of CVD. However, their review does not address the ongoing debate as to whether statin treatment is warranted for primary prevention of CVD, nor does it adequately address concerns regarding adverse effects of statins. We have therefore written a commentary which provides a broader perspective on the benefits versus harms of statin therapy. Our perspective of the literature is that non-adherence to statin treatment for primary prevention of CVD is justified because the meager benefits are more than offset by the extensive harms.

## Commentary

Over two decades ago, William Clifford Roberts, MD, editor of the American Journal of Cardiology, referred to statins as “*underused miracle drugs*”, which are to “*atherosclerosis what penicillin was to infectious disease*” [2]. He declared that statins “*have the capacity to prevent (coronary) events in the first place*”. Roberts expressed dismay, however, with the high rate non-adherence of patients with their statin regiment. He lamented that “*50% of patients placed on a lipid-lowering drug quit taking the drug in 1 year and only 25% still take the drug 2 years after it was started*.” Roberts urged physicians to “*convince patients that these miracle drugs … are the best anti-atherosclerotic insurance they can purchase*.”

Others have not been so sanguine regarding the benefits of statins for primary prevention of cardiovascular disease (CVD). For example, in an editorial in JAMA [3], Redberg and Katz provided statistics that may surprise most clinicians: “*a healthy man with elevated cholesterol will not live any longer if he takes statins*. *For every 100 patients with elevated cholesterol levels who take statins for 5 years*, *a myocardial infarction will be prevented in 1 or 2 patients*. *Preventing a heart attack is a meaningful outcome*. *However*, *by taking statins*, *1 or more patients will develop diabetes and 20% or more will experience disabling symptoms*, *including muscle weakness*, *fatigue and memory loss*.” Redberg and Katz concluded by stating that “*statins are not effective in improving length or quality of life when used for primary prevention*”.

It is in this context of decades of debate on the relative merits versus harms of statin treatment for prevention of CVD, and the well-documented low rate of adherence of patients to comply with their statin regimen, that Hope et al., [1] have published a review of predictors of statin adherence for primary prevention of CVD. Their review revealed that the majority of studies reported at least 40%, and as much as 80%, of patients did not comply fully with statin treatment recommendations. Their findings are consistent with a study of more than 140,000 elderly people which reported that 75% of those on statins for primary prevention stopped taking their medication [4], as well as a more recent study demonstrating that almost 90% of statin-treated patients discontinued treatment [5].

When the majority of those prescribed a statin show poor adherence, especially when their physician tells them it may prolong their lives and protect them from having a heart attack, it is important for investigators to consider all factors which may explain such a high rate of non-compliance. Patients who do not adhere to recommendations may have traits which contribute to non-compliance, such as poor dietary and lifestyle habits. The study by Hope et al., [1] quantified factors identified in randomized controlled trials (RCTs) and observational studies that were predictors of statin adherence. They reported that the primary predictors of statin adherence were limited to age, male gender, diagnosis of diabetes and hypertension, income, education, alcohol misuse and high BMI.

If, as Roberts [2] stated, statins were as effective in the treatment of CVD as penicillin is in the treatment of infectious disease, then Hope et al., [1] would be justified in stating “the extent to which these *(statin)* therapies will be effective is directly associated with the patient’s adherence to their treatment regimen”. However, we are not convinced that the balance between the benefits and harms of statins for primary prevention of CVD justifies the goal of improved adherence. First, numerous studies have reported an absence of benefits in primary prevention of CVD with statins (see [3, 6–9]). Second, the overall magnitude of beneficial effects is so slight as to be clinically insignificant. In accordance and based on a review of 19 trials (71,900 patients), the US Preventive Services Task Force (USPSTF) recently concluded that initiating statin use for the primary prevention of CVD events in adults 76 years and older without a history of CVD cannot be determined. They did recommend statin treatment if one or more risk factors are present, but as pointed out by Redberg and Katz [8], the absolute risk reductions (ARR) of CVD mortality and total mortality were only 0.43% and 0.40%, respectively. It is worth noting that these ARR figures were potentially augmented by the inclusion of data from patients taking statins for secondary prevention.

To support their perspective on the value of statins for primary prevention of CVD, Hope et al., [1] cited work from Taylor et al. [10], which was a summary of findings from primary prevention trials. Hope et al., [1] stated that the composite of fatal/non-fatal CVD events reported by Taylor et al. was reduced with statin treatment by a quarter (25%). This figure reflects the relative risk reduction (RRR), which is the ratio of the risk of CVD in the statin-treated group to that in the placebo-treated group. RRR is contrasted by the absolute risk reduction (ARR), which is the absolute magnitude of the difference in the incidence of CVD in the treated and untreated groups. The ARR reported by Taylor et al., for all CVD events was a 2.85% difference between statin- and placebo-treated groups. Thus, the same raw data can be expressed as a 25% benefit (RRR), as well as a 2.85% (ARR) benefit. To further compound the complexity of data presentation, Hope et al., noted that Taylor et al. had reported that the number needed to treat (NNT), a derivation of the ARR, was 56. This finding indicated that 56 people needed to be treated with a statin for 5 years to prevent a single adverse outcome. That the same raw data can be presented in multiple different forms (as RRR, ARR and NNT) can be confusing to readers [11, 12]. This is an important point because research has shown that patients, as well as physicians, tend to overestimate the value of treatment when they are provided the RRR, without the corresponding ARR [11–15]. For this reason, investigators have expressed concern at the appearance of inflated benefits when relative risk is presented without absolute risk [16–18].

Second, the 2.85% absolute risk benefit of statins is a composite of all fatal and non-fatal CVD events. This composite figure typically includes the combination of “soft” outcomes, such as revascularization procedures and “hard” events, such as CVD death, myocardial infarction and stroke. As pointed out by Abramson et al., [19], rates of revascularization are imprecise and potentially biased, because treatment decisions are unblinded, based to a large extent on total cholesterol and LDL levels. Abramson et al., [19] noted, for example, that 35% of “major vascular events” in the Cholesterol Treatment Trialists’ (CTT) Collaboration meta-analysis were coronary revascularization procedures. When “hard” events (major coronary events and stroke) were analyzed separately, the ARR was only 0.7% over the course of 5 years of treatment. According to Abramson, et al., in primary prevention of CVD for low risk patients, 167 people need to be treated with a statin for 5 years to prevent a single hard cardiovascular event. It is also worth noting that the one hard event statin treatment may prevent in 5 years may not be fatal, and could heal with minor future harms or none at all solely with diet and lifestyle changes [20].

A second issue particularly relevant to statin adherence, but not sufficiently addressed by Hope et al., [1] is that it is well-established that people terminate statin treatment because of perceived treatment-related adverse effects. Hope et al., [1] noted that there has been only 1 observational trial that quantified the relationship between side effects and adherence [21]. They mentioned the possibility that statin side effects could explain low adherence, but largely dismissed this possibility with the statement that “prior negative expectations may misattribute symptoms such as muscular pain (myalgia) to statin use”. The issue that statin-related myalgia is a “nocebo effect”, i.e., based on a person’s expectation of adverse effects, rather than the effects actually triggered by the drug, has been raised by some investigators [22–24]. However, the low rate of adherence to statins has been noted for decades, e.g.,[2], a finding that is inconsistent with the view that the spread of misinformation by an “internet cult” in recent years has influenced people to misattribute normal aches and pains to be statin side effects [22].

Adverse effects of statins are extensive, well-characterized and have been described in numerous reviews, at descriptive and mechanistic levels [25–30]. With regard to the issue of statin adherence, adverse effects may be considered to be of two categories. The first is an adverse effect which is perceived by the patient, and is a contributor to why statin adherence is so low. These perceived adverse effects include a high incidence of muscle pain [31–37], fatigue [38, 39], especially with exertion and exercise [40], idiopathic inflammatory myositis [41, 42], autoimmune myopathy [43–46], psychiatric and cognitive symptoms (depression, confusion, aggression, memory loss [47–55]), severe irritability [56], sleep disturbances [52], musculoskeletal disorders and injuries [57, 58], sudden sensorineural hearing loss [59] and gastrointestinal distress [60]. The second category includes later developing morbidities that may not be perceived by the users as a side effect, but are linked to statin treatment: Type-2 diabetes [27, 61–64], particularly in women [65–67], cancer [68–71], liver dysfunction and failure [72, 73], cataracts [74, 75], amyotrophic lateral sclerosis (ALS), ALS-like conditions and other central motor disorders, e.g., Parkinson’s disease and cerebellar ataxia [76–80], lupus-like syndrome [81], susceptibility to herpes zoster [82–84], interstitial cystitis [85], polymyalgia rheumatic [86], kidney injury [87, 88] and renal failure [89].

To understand why statin adherence is so low, clinicians and patients need to be provided with a comprehensive and unbiased assessment of the magnitude of the benefits of statin treatment, balanced against evidence of very real, adverse effects. It is in this context that we quote Redberg and Katz [8], who reviewed the issue of statin safety, effectiveness and adherence by concluding: “*It is incumbent on clinicians to be sure that before recommending that a patient take a daily pill that has multiple adverse effects*, *there is evidence that the medication will lead to a better quality of life*, *longer life*, *or both*. *Such evidence is lacking for statins in primary prevention*.”

In summary, we have integrated the literature on statin benefits versus harms, with the views of respected key opinion leaders, to conclude that non-adherence to statin treatment for primary prevention of CVD is justified because the meager benefits are more than offset by the extensive harms.
